# Conservation of Affinity Rather Than Sequence Underlies a Dynamic Evolution of the Motif-Mediated p53/MDM2 Interaction in Ray-Finned Fishes

**DOI:** 10.1093/molbev/msae018

**Published:** 2024-02-01

**Authors:** Filip Mihalič, Dahiana Arcila, Mats E Pettersson, Pouria Farkhondehkish, Eva Andersson, Leif Andersson, Ricardo Betancur-R, Per Jemth

**Affiliations:** Department of Medical Biochemistry and Microbiology, Uppsala University, BMC, Uppsala SE-75123, Sweden; Scripps Institution of Oceanography, University of California San Diego, La Jolla, CA 92093, USA; Department of Medical Biochemistry and Microbiology, Uppsala University, BMC, Uppsala SE-75123, Sweden; Department of Medical Biochemistry and Microbiology, Uppsala University, BMC, Uppsala SE-75123, Sweden; Department of Medical Biochemistry and Microbiology, Uppsala University, BMC, Uppsala SE-75123, Sweden; Department of Medical Biochemistry and Microbiology, Uppsala University, BMC, Uppsala SE-75123, Sweden; Department of Veterinary Integrative Biosciences, Texas A&M University, College Station, TX 77483, USA; Scripps Institution of Oceanography, University of California San Diego, La Jolla, CA 92093, USA; Department of Medical Biochemistry and Microbiology, Uppsala University, BMC, Uppsala SE-75123, Sweden

**Keywords:** protein evolution, affinity, sequence evolution, intrinsically disordered regions

## Abstract

The transcription factor and cell cycle regulator p53 is marked for degradation by the ubiquitin ligase MDM2. The interaction between these 2 proteins is mediated by a conserved binding motif in the disordered p53 transactivation domain (p53TAD) and the folded SWIB domain in MDM2. The conserved motif in p53TAD from zebrafish displays a 20-fold weaker interaction with MDM2, compared to the interaction in human and chicken. To investigate this apparent difference, we tracked the molecular evolution of the p53TAD/MDM2 interaction among ray-finned fishes (Actinopterygii), the largest vertebrate clade. Intriguingly, phylogenetic analyses, ancestral sequence reconstructions, and binding experiments showed that different loss-of-affinity changes in the canonical binding motif within p53TAD have occurred repeatedly and convergently in different fish lineages, resulting in relatively low extant affinities (*K_D_* = 0.5 to 5 μM). However, for 11 different fish p53TAD/MDM2 interactions, nonconserved regions flanking the canonical motif increased the affinity 4- to 73-fold to be on par with the human interaction. Our findings suggest that compensating changes at conserved and nonconserved positions within the motif, as well as in flanking regions of low conservation, underlie a stabilizing selection of “functional affinity” in the p53TAD/MDM2 interaction. Such interplay complicates bioinformatic prediction of binding and calls for experimental validation. Motif-mediated protein–protein interactions involving short binding motifs and folded interaction domains are very common across multicellular life. It is likely that the evolution of affinity in motif-mediated interactions often involves an interplay between specific interactions made by conserved motif residues and nonspecific interactions by nonconserved disordered regions.

## Introduction

**Fig. 1.**
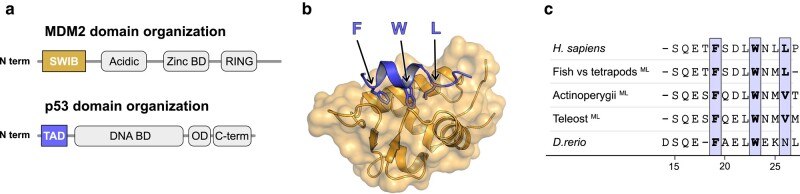
Structure of the human MDM2/p53TAD complex. a) Schematic domain architecture of MDM2 and p53. b) Crystal structure of the human complex between the SWIB domain of MDM2 (gold) and a peptide corresponding to the conserved binding motif in p53TAD (blue) (PDBid: 1ycr) (Kussie et al. 1996). Arrows highlight the conserved hydrophobic triad where the residues are shown as sticks. c) Sequence alignment of reconstructed and extant canonical MDM2-binding motifs of p53TAD. To facilitate comparison, the site numbering used here follows the human p53TAD-binding motif, from Ser_15_ to Pro_27_. The previously reconstructed maximum likelihood sequence for the most recent common ancestor of bony fishes and tetrapods (Mihalič et al. 2023) is indicated in the alignment as “fish versus tetrapods.”

The interaction between p53 and its regulator the ubiquitin ligase MDM2 is a well-studied system due to the central role of p53 as a master cell cycle transcription factor (Vousden and Prives 2009; Levine 2020). The interaction between the proteins is mediated by a conserved binding motif in the intrinsically disordered N-terminal transactivation domain (TAD) of p53 and a SWIB domain in MDM2 (Fig. 1). The interaction leads to MDM2-catalyzed ubiquitination and proteasomal degradation of p53. Phosphorylation of p53TAD increases affinity for the general transcriptional coactivators CBP/p300 and decreases affinity for MDM2 (Ferreon et al. 2009; Teufel et al. 2009; Lee et al. 2010). This phosphorylation-dependent activation of p53 leads to the transcription of several genes, including the gene-encoding MDM2, resulting in a negative feedback loop and fluctuating low levels of p53 under normal conditions. Bioinformatic and experimental analyses suggested that the p53–MDM2 regulation is an ancient mechanism, already present in the common ancestor of all metazoans (Lane et al. 2010; Lane and Verma 2012; Siau et al. 2016; Zhang et al. 2022). We recently investigated the deep evolution of affinity between p53TAD and MDM2 and found that the interaction between the conserved binding motif in p53TAD and MDM2 SWIB is relatively weak in invertebrates, ranging from not measurable to *K_D_* = 15 μM in the mollusk bay mussel (*Mytilus trossulus*) (Mihalič et al. 2023). Furthermore, the affinity was very low (*K_D_* = 250 μM) in the jawless vertebrate arctic lamprey (*Lethenteron camtschaticum*), whereas in other vertebrates such as human (*Homo sapiens*) and chicken (*Gallus gallus*), the affinity of the interaction is in the 100 nM range. We found that a reconstructed p53TAD from the ancestor of fishes and tetrapods bound with high affinity to its contemporary MDM2 as well as to extant MDM2s from human and zebrafish (*Danio rerio*). However, the extant *D. rerio* interaction between a peptide corresponding to the conserved binding motif in p53TAD*_D.rerio_* (residues 15 to 26) and MDM2*_D.rerio_* displayed no affinity in our binding assay due to an apparent insertion of an asparagine residue into the binding motif at position 26, which destabilizes the binding interface. The addition of leucine (p53TAD^15–27^), the next residue of the motif, partially restored the canonical hydrophobic triad in the motif (F_19_xxxW_23_xxN_26_L_27_) and resulted in measurable affinity but still approximately 20-fold weaker than the p53TAD-MDM2 interactions of human, chicken, and the fishes/tetrapods ancestor. Apart from the Asn_26_ insertion, the lower affinity of the *D. rerio* interaction is also due to the absence of Thr_18_, a helix N-cap residue present in most vertebrate p53TADs and whose phosphorylation decreases affinity for MDM2 and increases affinity for CBP/p300 (Sakaguchi et al. 2000; Teufel et al. 2009; Lee et al. 2010).

In the present study, we further investigate this intriguing apparent loss of affinity in *D. rerio* p53TAD by performing a comprehensive phylogenetic analysis of p53TAD for ray-finned fishes (Actinopterygii), combined with experiments to determine the affinity between p53TAD and the SWIB domain of MDM2 from representative extant fish families, and for reconstructed p53TAD and MDM2 variants from ancestral fishes. A fascinating picture emerges, in which loss-of-affinity mutations in the conserved MDM2-binding motif of p53TAD have occurred several times in different fish lineages. We also find that the loss of affinity in p53TAD is compensated by the C-terminal flanking region of the conserved motif. Our analyses show how regions outside of the conserved binding motif promote binding and contribute to an extraordinarily dynamic evolution of the p53–MDM2 interaction. These findings demonstrate a high plasticity of motif-mediated interactions involving transactivation domains such as p53TAD and the uncertainty of bioinformatic inference of binding without experimental validation. It is likely that similar mechanisms of affinity modulation by flanking regions are widespread in motif-mediated interactions.

## Results

### Phylogenetic Analysis of Fish p53TAD

**Fig. 2.**
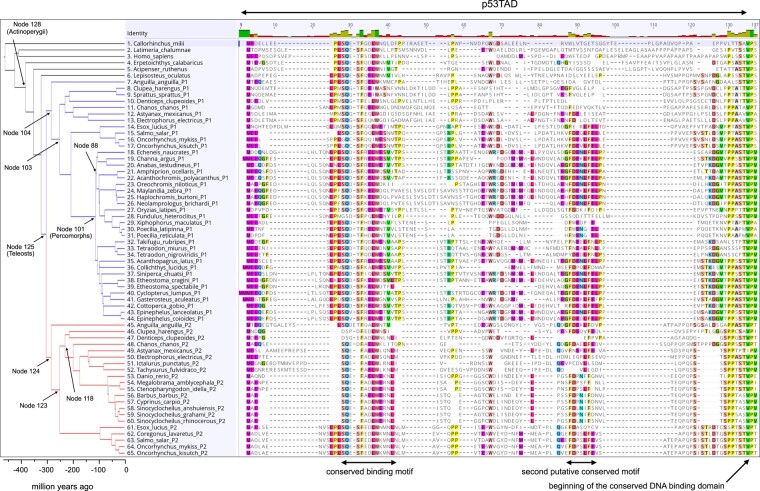
Sequence alignment of p53TAD mapped onto the fish phylogeny. We included 51 extant species representing some of the major lineages of fishes and for which high-quality sequencing data were available. The blue and red branches represent the split between the 2 paralogs p53TAD_P1_ and p53TAD_P2_, respectively, in teleosts (node 125). After the split, p53TAD_P1_ and p53TAD_P2_ have evolved in parallel in each lineage. Node numbers refer to the ancestral reconstruction analysis (supplementary Spreadsheet File S1, Supplementary Material online).

To investigate the evolution of p53TAD in ray-finned fishes, we collected p53 sequences from 51 species of Actinopterygii. We also included 3 other vertebrates as outgroups in the sequence alignment: human, West Indian Ocean coelacanth (*Latimeria chalumnae*), and Australian ghostshark (*Callorhinchus milii*). In several species, we found 2 distinct p53 proteins, and phylogenetic analyses suggested that they are paralogs originating in the third whole-genome duplication in teleosts (Teleostei) (Glasauer and Neuhauss 2014), the dominant clade within Actinopterygii (Fig. 2). In salmonids (Salmonidae) that underwent a fourth genome duplication (Lien et al. 2016), we found 3 paralogs of p53. In cases where the phylogeny of the 2 p53 paralogs was uncertain from sequence analysis alone, such as European eel (*Anguilla anguilla*), which diverged early from other teleost fishes, the identity of the paralog was confirmed by its local chromosomal context, assessed from the genome databases at ENSEMBL and NCBI. Paralogs assigned to “Class II” (also referred to as paralog 2) were flanked by *gps2* in the sense direction, on the upstream side and an antisense sequence of an E3 ubiquitin–protein ligase followed by a *capga* homolog on the downstream side. “Class I” paralogs (paralog 1) had variable downstream contexts but were always flanked by *slc2a4* in antisense direction on the upstream side. The sequence variation within p53TAD is large and with many insertions and deletions (indels), which makes accurate sequence alignment and a reliable gene tree inference challenging. Instead, we employed a reference species phylogeny (Betancur-R et al. 2017; Hughes et al. 2018) as the analytical framework. This approach not only mitigated these challenges but also facilitated the identification of ancestral changes in the amino acid sequence across different branches of the fish tree of life.

Amino acid sequences were first aligned using Clustal Omega (Sievers et al. 2011), followed by manual alignment curation. Indeed, a high-confidence alignment of the entire p53TAD is difficult to obtain due to the extensive changes in most parts of the sequence, including both point mutations and indels. Nevertheless, the obvious limitations of the alignment also demonstrate the very dynamic evolution of p53TAD that has occurred along different lineages (Fig. 2). The p53TAD alignment was used together with the reference fish phylogeny to reconstruct the conserved binding motif in p53TAD at different ancestral nodes using maximum likelihood model in MEGAX (supplementary Spreadsheet S1, Supplementary Material online). Note that we show a reconstruction of the entire p53TAD in the Supplementary material, but it is only the 12-residue canonical binding region, which interacts with MDM2, as well as the N-terminal region of the DNA-binding domain that served as a reference point, that can be reconstructed with any confidence (supplementary Text File S1, Supplementary Material online presents a list of posterior probabilities for each position in each node). In addition, a second putative transactivation domain motif is conserved among most teleosts (Fig. 2). The reconstructed sequence of this second motif for the ancestral teleost is FDEKLFE (supplementary Spreadsheet S1, Supplementary Material online), and the consensus motif for the teleost sequences in Fig. 2 is FDENLFE (determined with Jalview; Waterhouse et al. 2009). There is no sequence similarity between this motif and the second motif in mammalian p53TAD. Thus, most likely the second fish p53TAD motif has evolved independently from mammalian p53TAD2. A reconstruction of ancestral maximum likelihood sequences was also performed for the folded SWIB domain of MDM2 (supplementary fig. S1, Spreadsheet S2, and Text File S2, Supplementary Material online). To account for uncertainty in the reconstruction, we performed experiments with alternative reconstructed variants (AltAll) where residues with a probability below 0.9 were replaced by the second most likely residue (Eick et al. 2017) (supplementary Spreadsheets S3 and S4, Supplementary Material online). Based on the available sequence data and the reference phylogeny used in the reconstruction, there are several crucial historical events of molecular evolution that can be established with high confidence.

### Evolution of the Binding Affinity between p53TAD Canonical Motif and MDM2 in Fishes

**Fig. 3.**
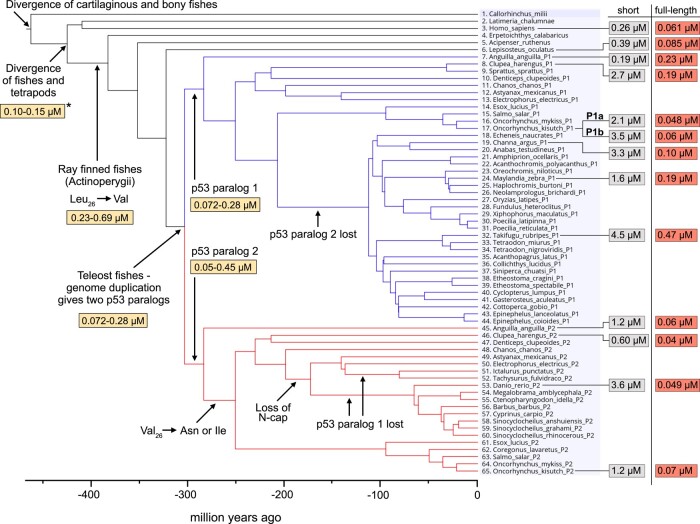
Affinities of p53TAD/MDM2 interactions. Affinities (*K_D_* values) of extant and ancient p53TAD^15–27^/MDM2 for different fishes mapped onto the phylogenetic tree. Key events that are discussed in the text are shown. Affinities for the reconstructed p53TAD^15–27^/MDM2 are highlighted in yellow, the affinities of p53TAD^15–27^/MDM2 for present-day fish species in gray and for the p53TAD^full-length^/MDM2 in red. See Table 1 and supplementary table S1, Supplementary Material online for errors. The affinity at the node representing the divergence of fishes and tetrapods, marked with a star, was determined previously (Mihalič et al. 2023).

To trace the evolution of affinity of the p53TAD-binding motif across Actinopterygii species, we expressed and purified reconstructed and extant MDM2 SWIB domains and measured the affinity using a fluorescence polarization-based method. We included 4 ancient (ancestral Actinopterygii, teleost, paralog 1, and paralog 2, respectively) and 12 extant MDM2s and p53TAD peptides containing the conserved binding motif (corresponding to human p53 residues 15 to 27, p53TAD^15–27^; Fig. 1). The extant MDM2 and p53TAD sequences were from 8 fishes representing different evolutionary lineages in the phylogenetic tree and 4 paralogs from 3 of these species. Additionally, we included the human MDM2/p53TAD interaction for reference (Table 1; Fig. 3; supplementary Spreadsheet S5, Supplementary Material online).

**Table 1:** Affinities for extant MDM2/p53TAD interactions

Species	p53TAD^15–27^ native	*K_D_* (μM) native^a^	*K_D_* (μM)^b^ MDM2*_H.sapiens_*	*K_D_* (μM)^c^ p53TAD*_H.sapiens_*^15–27^	*K_D_* (μM)^d^ p53TAD^full-length^ native
*Homo sapiens* (human)	SQET**F**SDL**W**KL**L**P	0.26 ± 0.02	-	-	0.061 ± 0.003 0.10 ± 0.003^e^
*Lepisosteus oculatus* (spotted gar)	SQESFQELWNMVI	0.39 ± 0.04	0.121 ± 0.005	0.37 ± 0.014	0.085 ± 0.005
*Anguilla anguilla* (European eel) p53TAD_P1_	SQESFQELWKMMC	0.19 ± 0.02	0.013 ± 0.001	0.9 ± 0.1	0.23 ± 0.02
*Anguilla anguilla* p53TAD_P2_	SQDETFQALWNTVT	1.2 ± 0.2	0.77 ± 0.02	…	0.06 ± 0.01
*Clupea harengus* (Atlantic herring) p53TAD_P1_	SQGTFDEIWASNF	2.6 ± 0.2	1.25 ± 0.04	1.8 ± 0.3	0.19 ± 0.04
*Clupea harengus* p53TAD_P2_	DSPDFEDLWNSIV	0.60 ± 0.03	0.29 ± 0.02	…	0.04 ± 0.01
*Oncorhynchus kisutch* (coho salmon) p53TAD_P1a_	GQCSFQQLWESNM	2.1 ± 0.2	3.15 ± 0.06	0.78 ± 0.1	0.048 ± 0.005
*Oncorhynchus kisutch* p53TAD_P1b_	SQGSFQQLWETNM	3.5 ± 0.1	5.15 ± 0.09	…	0.06 ± 0.01
*Oncorhynchus kisutch* p53TAD_P2_	SQESFEDLWKMNL	1.16 ± 0.07	0.79 ± 0.03	…	0.07 ± 0.01
*Channa argus* p53TAD_P1_	SQDSFRELWESVV	3.3 ± 0.4	1.1 ± 0.3	2.1 ± 0.2	0.10 ± 0.03
*Maylandia zebra* p53TAD_P1_	SQDSFKELWDQLP	1.6 ± 0.1	0.27 ± 0.04	1.7 ± 0.4	0.19 ± 0.02
*Takifugu rubripes* (Japanese puffer) p53TAD_P1_	SQDTFQDLWENVA	4.5 ± 0.8	1.7 ± 0.1	1.1 ± 0.2	0.47 ± 0.03
*Danio rerio* (zebrafish) p53TAD_P2_	SQEFAELWEKNL	3.6 ± 0.3	4.33 ± 0.05	0.79 ± 0.05	0.049 ± 0.008

*K_D_* values were measured using a fluorescence polarization-based displacement assay. The complex between MDM2 and FITC-labeled p53TAD_human_^15–27^ was displaced by unlabeled p53TAD^15–27^ or full-length p53TAD^full-length^ from different species and the IC_50_ value was used to calculate *K_D_*.

^a^Native *K_D_* refers to the interaction between p53TAD and MDM2 from the same species.

^b^ *K_D_* for the interaction between MDM2*_H.sapiens_* and the respective fish p53TAD.

^c^ *K_D_* for the interaction between p53TAD*_H.sapiens_*^15–27^ and the respective fish MDM2.

^d^ *K_D_* for the interaction between a full-length p53TAD and MDM2 from the same species.

^e^From Åberg et al. (2018).

The previous reconstruction of the p53TAD canonical binding motif from the common ancestor of fishes/tetrapods (Mihalič et al. 2023) suggested the presence of all three hydrophobic residues (in bold) that point into the binding pocket and make interactions with MDM2 in human p53TAD: S_15_Q_16_E_17_T_18_**F_19_**S_20_D_21_L_22_**W_23_**K_24_L_25_**L_26_** (Fig. 1). While the Phe_19_ and Trp_23_ are strictly conserved among Actinopterygii, there is considerable variation with regard to the third hydrophobic residue (Leu_26_) and the following position 27, which was not included in the previous reconstruction of p53TAD_fishes/tetrapods_ (Mihalič et al. 2023). Here, we included both residues in the experiments since residue 27 contributes to affinity in p53TAD peptides derived from extant *D. rerio* (Mihalič et al. 2023). In the p53TAD_Actinopterygii_^ML^ (superscript ML or AltAll indicate most likely and least likely reconstructed sequences, respectively) motif, the ancestral Leu_26_ in p53TAD_fishes/tetrapods_^ML^ had been replaced by Val_26_. This, along with 2 other substitutions (Fig. 1c), resulted in an affinity of 690 nM toward reconstructed maximum likelihood MDM2_Actinopterygii_^ML^ (Fig. 3; supplementary table S1, Supplementary Material online). This affinity is lower than the affinity between the ancestral fishes/tetrapods proteins (*K_D_* = 70 to 200 nM). Indeed, Leu_26_ → Val is a loss-of-affinity mutation, as shown by the 5- to 10-fold increase in affinity of p53TAD_Actinopterygii_^ML^ and p53TAD_teleost_^ML^ peptides with the reverse Val_26_ → Leu mutation (supplementary table S1, Supplementary Material online). The reconstructed AltAll sequences for the ancestral Actinopterygii complex display slightly higher affinities than the maximum likelihood variants, yielding a *K_D_* = 230 nM for the MDM2_Actinopterygii_^AltAll^ and p53TAD_Actinopterygii_^AltAll^ interaction.

Val_26_ is conserved in p53TADs of extant nonteleost Actinopterygii and in paralog 1 of most percomorphs (18 to 44 in the gene tree; Fig. 2). The reconstructed p53TAD at node 125, representing an ancestral teleost p53TAD before the genome duplication, has a posterior probability of 0.60 for Val_26_ and 0.39 for Ile_26_ (supplementary Spreadsheet S3, Supplementary Material online; Fig. 2). Despite this uncertainty, a comparison of affinities (Fig. 3; supplementary table S1, Supplementary Material online) of the peptides representing the ancestral p53TAD_teleost_, p53TAD_P1_, and p53TAD_P2_ (see below) suggests that higher affinity of the motif possibly evolved in the ancestral teleost as compared to the ancestral ray-finned fish by a change from Val_26_Thr(Ala)_27_ to Val(Ile)_26_Met(Leu/Val)_27_, with AltAll amino acids in parenthesis. However, if the alanine was present at position 27 in p53TAD_Actinopterygii_, the ancestral Actinopterygii and teleost complexes were of similar affinity.

The teleost-specific whole-genome duplication created 2 paralogous p53 genes with separate evolutionary histories (Fig. 2). The reconstructed last common ancestors of paralog 1 (p53TAD_P1_, node 104) and paralog 2 (p53TAD_P2_, node 124) are both very similar to p53TAD_teleost_, and the three reconstructed binding motifs have the same ambiguity with regard to Val_26_/Ile_26_. However, since Val_26_ is spread over the p53TAD_P1_ clade defined by node 101 and is also present in p53TAD_P2_ of *A. anguilla*, the most likely scenario is that Val_26_ is ancestral for p53TAD_P1_, p53TAD_P2_, and p53TAD_teleost_ and that Ile26 is derived later in certain lineages. Assuming that Val_26_ is ancestral, this residue is largely conserved in most percomorphs (Percomorpha; species 18 to 44) in p53TAD_P1_. Position 27 is very uncertain for p53TAD_teleost_, with around 25% probability for either Met, Leu, or Val, reflecting the different residues at this position in extant fishes. Thus, the ancestral Val_26_ and the uncertain residue at position 27 have been replaced by different residues in nonpercomorph teleosts, a paraphyletic group encompassing extant species 8 to 17, including Atlantic herring (*Clupea harengus*; Asn_26_Phe_27_), denticle herring (*Denticeps clupeoides*; Leu_26_Gly_27_), electric eel (*Electrophorus electricus*; Ile_26_Gly_27_), and the salmonids (Asn_26_Met_27_) (supplementary fig. S2, Supplementary Material online). In p53TAD_P2_, we observe further intriguing modifications at the end of the binding motif. Either in ancestral node 123, after the divergence of the p53TAD_P2_ branch leading to *A. anguilla*, or, alternatively, at least three times in separate branches (supplementary fig. S3, Supplementary Material online), there is a change of Val_26_ → Asn. This mutation to Asn_26_ is particularly notable since it has also occurred in certain p53TAD_P1_ lineages (salmonids and European sprat [*Sprattus sprattus*]), thus representing a recurrent loss-of-affinity mutation. Extant *A. anguilla* p53TAD_P2_ retains Val_26_ either as a result of back mutation or conservation. *C. harengus* (Ile_26_Val_27_) and milkfish (*Chanos chanos*) p53TAD_P2_ (Ile_26_Thr_27_) have gained subsequent mutations at the end of the motif. We note here that it is difficult to judge whether the described changes at positions 26 to 27 are due to point mutation or indels, as several residues in the region are different among extant fishes, obscuring the historical order of events (supplementary figs. S2 and S3, Supplementary Material online).

To further rationalize determinants of affinity, we aligned the p53TAD-binding motifs and ranked them based on *K_D_* values for native MDM2 (supplementary fig. S4a, Supplementary Material online) and for MDM2*_H.sapiens_* (supplementary fig. S4b, Supplementary Material online), which clearly showed that nonconserved residues such as those at position 24 and in particular 25 can affect affinity. To some extent, in the case that the residue in position 26 is not hydrophobic, position 27 will also influence binding. Interestingly, Met_25_ is recurrent in high-affinity p53TAD peptides, in particular with Leu_26_, Ile_26_, or Met_26_ in the adjacent position and also with Val_26_. Met_25_ was likely present in the ancestral p53TAD_Actinopterygii_ and p53TAD_teleost_. Its subsequent mutation in node 103, i.e. all p53TAD_P1_ except *A. anguilla*, and in most p53TAD_P2_ (except the salmonids) is another example of a recurrent loss-of-affinity change. The N-cap at position 18 stabilizes the helix formed by the conserved 12-residue motif upon binding to MDM2, via a hydrogen bond to Asp_21_ in the human complex (Kussie et al. 1996). The ancestral p53TAD_teleost_ Ser_18_ was likely replaced in node 118 (p53TAD_P2_) by Asp_18_, which could potentially also function as N-cap (Fig. 2). However, Asp_18_ cannot act as a phosphorylation switch regulating MDM2 and CBP/p300 affinity and was subsequently deleted in the common ancestor of otophysans (Otophysi; p53TAD_P2_, species 49 to 60, including *D. rerio*). Experiments using different p53TAD*_D.rerio_* peptides previously demonstrated that this is another loss-of-affinity mutation (Mihalič et al. 2023).

We also observed complete gene loss in some taxa. In fact, p53_P1_ was apparently lost twice, once in an ancestor of cypriniformes (species 53 to 60, including *D. rerio*) and once in the lineage leading to noncypriniform otophysans (species 51 to 52). The gene encoding p53_P2_ was lost in the common ancestor of the percomorph lineages Ovalentaria, Carangaria, and Anabantaria (species 18 to 31; node 88). Thus, we found both paralogs, p53_P1_ and p53_P2_, in a limited number of species including *A. anguilla*, *C. harengus*, and the salmonid family. Clearly, an alternative explanation is that sequence data are incomplete and that we miss some paralogs. However, the clustering of sequences (or lack of sequences) in the phylogenetic tree supports a scenario where the paralogs were indeed lost, as it is unlikely that potential assembly errors would be systematic in such fashion. Furthermore, we investigated chromosome locations surrounding the characteristic flanking genes but were unable to detect additional p53 homologs.

Intriguingly, the affinities of the extant complexes were generally relatively weaker across the fish tree between native p53TAD^15–27^ binding motifs and MDM2 (*K_D_* = 0.2 to 5 μM), as compared to the ancestral interactions (0.05 to 0.7 μM) and the human interaction (*K_D_* = 0.26 μM) (Fig. 3). For a direct comparison of the different fish p53TADs and MDM2s, we also measured (i) the affinity between p53TAD_human_^15–27^ and the fish MDM2s and (ii) the affinity between fish p53TADs and human MDM2. The trend was the same with relatively low affinities for fish p53TADs as compared to the human interaction. Moreover, the affinities were in general slightly higher when either human p53TAD or human MDM2 was in the complex as compared to the native fish interaction (Table 1).

Our experimental results and the sequence alignment of fish p53TADs suggest an intriguing scenario: the affinity between the canonical binding motif in p53TAD and MDM2 appears to first have decreased in the ancestral Actinopterygii p53TAD (Leu_26_→Val) and then increased by changes at position 27 to a large hydrophobic residue in the ancestral p53TAD_teleost_ and then—for example, via deletion of Asp_18_, changes at Met_25_, or Val_26_ → Asn—decreased again such that 10 of the 12 investigated present-day canonical motifs display *K_D_* values in the range of 0.6 to 4.5 μM across teleost fishes. Importantly, due to poor conservation of the p53TAD outside of the conserved binding motif, we were not able to reconstruct full-length ancestral p53TAD and can therefore only make conclusions regarding the interaction between ancestral MDM2 and the conserved motif.

### Flanking Regions Increase the Affinity between p53TAD and MDM2

**Fig. 4.**
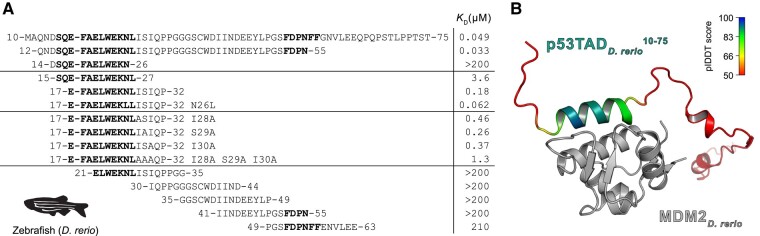
Affinities between p53TAD peptides and MDM2 from *D. rerio*. a) The overlapping peptides tiling a 51-residue stretch of p53TAD*_D.rerio_*. To facilitate comparison, the numbering starts from the conserved motif and the residues of human p53TAD, F_19_xxxW_23_xxL_26_. With that in mind, the p53TAD^10–75^ in fact contains the whole TAD region of *D. rerio* and corresponds to p53TAD^full-length^. *K_D_* values for the peptide fragments were determined by the fluorescence polarization assay. Distal residues and Ile_28_Ser_29_Ile_30_ contribute to the affinity for MDM2. Both conserved motifs are in bold. The zebrafish silhouette was obtained from phylopic.org and is under CC0 1.0 DEED licence (https://creativecommons.org/publicdomain/zero/1.0/). b) ColabFold prediction of the interaction between *D. rerio* MDM2 and p53TAD^10–75^. The MDM2 is in gray, and the p53TAD^10–75^ is colored according to the pIDDT, where blue indicates highest and red lowest prediction confidence (pIDDT < 50).

Despite the relatively low affinity, MDM2 is crucial for regulating p53 levels in *D. rerio* as demonstrated by knockout experiments (Chua et al. 2015). Therefore, we expressed, purified, and subjected a longer p53TAD construct from *D. rerio* to binding experiments (Fig. 4a). Surprisingly, we found that this construct, p53TAD_*D.rerio*_^10–75^, displayed more than 70-fold higher affinity (*K_D_* = 49 nM) than the p53TAD^15–27^_*D.rerio*_ and even higher affinity than human and chicken p53TAD/MDM2 interactions. For the human complex, a corresponding p53TAD_human_^13–61^ increased the affinity only 1- to 4-fold (depending on ionic strength) compared to the shorter p53TAD_human_^15–29^, as determined by kinetic experiments (Åberg et al. 2018) and confirmed in this study (4-fold by fluorescence polarization; Fig. 3 and supplementary Spreadsheet S6, Supplementary Material online).

To pinpoint the residue(s) contributing to the higher affinity, six overlapping peptides corresponding to p53TAD^12–63^_*D.rerio*_ were used in fluorescence polarization competition experiments. The peptide p53TAD^17–32^_*D.rerio*_ containing the conserved binding motif and the additional residues ISIQP partially recapitulated the affinity of full-length p53TAD^full-length^_*D.rerio*_ (*K_D_* = 180 nM compared to 49 nM, respectively; Fig. 4a). The remaining shorter peptides displayed negligible affinity. We additionally observed that the UniProt protein sequence database contains 2 different *D. rerio* p53 sequences under entries G1K2L5 and P79734, the latter marked as canonical sequence and lacking the Ser_29_. This alternative p53TAD^17–32^_*D.rerio*_ (P79734), ending with IIQPP, bound slightly weaker (*K_D_* = 580 nM) than ISIQP. We then replaced each of the residues Ile_28_Ser_29_Ile_30_ with Ala. The combined effect of each point mutation (7.5-fold reduced affinity) was identical within experimental error to the triple Ala mutant (AAAQP) (7.7-fold), showing that several residues close to the motif contribute additively to the increase in affinity (Fig. 4a).

To validate the *K_D_* values determined by competition experiments and gain insight into the thermodynamic parameters of the interaction, we performed ITC experiments with MDM2*_D.rerio_* and a short as well as the full-length p53TAD construct (p53TAD_*D.rerio*_^17–32^ and p53TAD_*D.rerio*_^full-length^, respectively). The results were in excellent agreement with fluorescence polarization data (*K_D_* = ∼300 and 100 nM for short and full-length p53TAD constructs, respectively; supplementary fig. S5, Supplementary Material online). The binding of both the short motif and full-length p53TAD is mainly enthalpy driven, but the differences between the thermodynamic parameters are too small to draw any conclusions regarding the higher affinity of full-length p53TAD.

To rationalize this result, we performed ColabFold (Mirdita et al. 2022) predictions of the *D. rerio* MDM2/p53TAD^10–75^ complex and observed that the algorithm predicted an extension of the p53TAD-binding helix including residues ISIQP (Fig. 4b). Such an extended helix is not predicted for the human complex (supplementary fig. S6, Supplementary Material online). Importantly, in p53TAD*_H.sapiens_*, the canonical motif is immediately followed by a helix-breaking proline (position 27). Previous experiments on the human complex involving a Pro_27_ → Ser substitution showed that the helicity increased and the affinity increased 10- to 100-fold (Zondlo et al. 2006), an effect we confirmed here with a Pro_27_ → Ala variant of p53TAD_*H.sapiens*_^17–32^ (supplementary fig. S7 and Spreadsheet S6, Supplementary Material online).

**Fig. 5.**
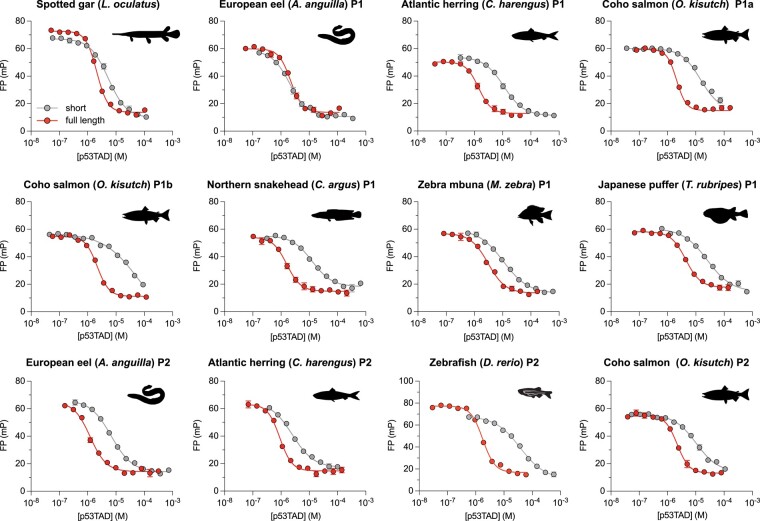
Affinity of short and full-length p53TAD of different extant fishes measured by fluorescence polarization competition experiments. Affinity is higher for full-length p53TAD constructs (Fig. 2; supplementary Spreadsheet S5, Supplementary Material online) as compared to the short p53TAD^15–27^, often by an order of magnitude, with the exception of *A. anguilla* p53TAD_P1_. FP, fluorescence polarization in millipolarization units (mP). Animal silhouettes were obtained from phylopic.org and are under CC0 1.0 DEED licence (https://creativecommons.org/publicdomain/zero/1.0/).

Inspired by the data for the *D. rerio* complex, we attempted the expression and purification of longer p53TADs from the same extant fishes where we already measured the affinity for the shorter p53TAD^15–27^ constructs, to assess whether the high affinity of the longer p53TAD is a universal phenomenon across the fish clade. These longer p53TAD^full-length^ constructs consisted of the entire region from the first amino acid of p53 to the beginning of the conserved DNA-binding domain, which is the C-terminal part of the alignment (Fig. 2; supplementary Spreadsheet S5, Supplementary Material online). We were able to express and purify full-length p53TADs for all 12 fish interactions. Remarkably, in 11 cases, the affinity increased considerably (4- to 73-fold; Table 1 and Figs. 3 and 5) resulting in *K_D_* values in the same range as that of the human complex (∼100 nM). Inspection of the flanking C-terminal sequences shows a low degree of conservation suggesting that different combinations of amino acid residues can promote increased affinity (supplementary fig. S8, Supplementary Material online). Additionally, the ColabFold predictions identified extended helices for several but not for all cases of bound p53TADs from different fishes suggesting alternative mechanisms for increasing the affinity, as compared to *D. rerio* p53TAD/MDM2 (supplementary fig. S9, Supplementary Material online). As we could not reconstruct ancestral full-length p53TAD sequences, we cannot conclusively say how the interaction evolved outside of the conserved interaction motif. However, given the fact that the MDM2 and p53 genes are present across the animal kingdom and apparently functional in the majority of its clades (Levine 2020), we conclude that the ancestral complex likely possessed similar “functional affinity” as extant complexes, which cluster around ∼100 nM (Fig. 4). The alternative scenario where the affinities for the present-day p53TAD/MDM2 complexes evolved to be radically different than the ancestral complex, while remaining similar among themselves is much less likely.

## Discussion

The gene encoding an ancestral p53-like protein was duplicated twice in an early vertebrate around 450 million years ago (Mya), before the divergence of cartilaginous fishes (Chondrichthyes), bony fishes (Osteichthyes), and tetrapods (Tetrapoda), in 2 whole-genome duplications (McLysaght et al. 2002; Vandepoele et al. 2004; Dehal and Boore 2005; Smith et al. 2013). From these events, 3 paralogs remain in extant vertebrates, namely p53, p63, and p73. Vertebrate p53 evolved to become a key cell cycle regulator, keeping its ancestral regulation by the ubiquitin ligase MDM2 (Vousden and Prives 2009; Levine 2020). The evolution of the molecular interaction between a conserved motif in the transactivation domain of p53-family proteins and MDM2 has been very dynamic with reduced affinity in some animal lineages (Mihalič et al. 2023) and even complete loss of the interaction domains (Åberg et al. 2017). We previously reconstructed p53TAD and MDM2 from the last common ancestor of fishes and tetrapods (Fig. 1). In the present study, we follow the branches down the Actinopterygii lineages to investigate the evolution of affinity between p53TAD and MDM2 in fishes. Interestingly, loss-of-affinity mutations in the binding motif have been selected for on several occasions in different fish lineages: Leu_26_ → Val (400 Mya), Met_25_ → Ser (250 Mya, p53TAD_P1_), Val_26_ → Asn (250 Mya or later, p53TAD_P2_), deletion of an N-cap residue (180 to 200 Mya, p53TAD_P2_), and Ile_26_ → Asn (40 to 90 Mya, p53TAD_P1_) (Fig. 2; supplementary figs. S2 and S3, Supplementary Material online). In addition, complete loss of the paralogs occurred: p53_P1_ (70 to 180 Mya, likely in 2 separate branches) and p53_P2_ (120 to 210 Mya).

In a larger context, a wealth of data has demonstrated the importance of short binding motifs for protein–protein interactions (Tompa et al. 2014). The interaction between a typical binding motif and an interaction domain usually involves a “short linear interaction motif” of only 3 to 10 amino acid residues, which provides a functional affinity with *K_D_* values in the nanomolar–micromolar range. However, recent data suggested that flanking disordered regions of the binding motif may modulate affinity (Bugge et al. 2020; Theisen et al. 2021; Karlsson et al. 2022). Activation domains are enriched in hydrophobic and negatively charged residues, where the latter likely contribute to exposure of hydrophobic motifs (Staller et al. 2022). While certain residues (aromatic, Leu) are vital, it has been proposed that the composition, rather than the exact sequence, plays a more important role for transcriptional activity (Zarin et al. 2017; Sanborn et al. 2021; Staller et al. 2022). Contrary to that, our data show that details in the motif could indeed matter. For example, it would be hard to predict that *A. anguilla* p53TAD_P1_ is the sole high-affinity extant teleost motif among those included in the study, with a positively charged Lys next to Trp_23_ and a Met at position 26.

Our present work on the p53/MDM2 interaction in ray-finned fishes provides a striking example of the potential role of rapidly evolving flanking regions, where the C-terminal flanking regions from 11 out of 12 different Actinopterygii p53TADs increase the affinity 4- to 73-fold as compared to the canonical conserved binding motif. In the human p53TAD/MDM2 complex, with a relatively high affinity for the canonical motif, only a 4-fold increase was observed. Indeed, also for the fish complexes, the lowest influence from flanking regions was found in the 2 cases with highest affinity of the motif (Fig. 3). Furthermore, the lack of correlation between *K_D_* values for the motif and full-length p53TAD (*R*^2^ = 0.014) reinforces the notion that the flanking regions make affinities more similar (supplementary fig. S10, Supplementary Material online). Thus, if ancestral full-length p53TAD/MDM2 complexes had a similar affinity as the extant ones, it is reasonable to envision a coevolution between the conserved motif and the flanking regions to maintain this “functional affinity.” It is important to note that apart from MDM2, several other proteins interact with p53TAD (Levine 2020). Thus, the dynamic evolution of the p53TAD sequence, and its interaction with MDM2, is likely influenced by other proteins and interactions that may fine-tune the regulation of p53.

Clearly, residues 25 to 27 influence affinity, and this region samples different combinations of residues over evolutionary time, possibly contributing to adaptation when a new niche is explored. These and other loss-of-affinity or gain-of-affinity mutations could be compensated by additional changes in the C-terminal flanking region to maintain affinity. Since reconstruction of the C-terminal part of p53TAD is not possible, we can only speculate about the order of events. One possible scenario is that the extensive mutations occurring in the C-terminal part first led to increased affinity between the proteins, resulting in a suboptimal but manageable high affinity. Compensatory mutations in the binding motif could then restore a lower affinity. It is harder to envision initial loss-of-affinity mutations in the binding motif since a 10-fold decrease in affinity would likely lead to severe effects on p53 regulation, impaired fitness, and elimination of the variant by negative selection. Initial mutations leading to changes in the concentration of proteins could also be the cause of subsequent mutations in the binding motif of p53TAD to restore fitness. In this context, it is interesting to consider Pro_27_, which is present in extant mammals, birds, and frogs (Mihalič et al. 2023), and therefore likely present in the ancestral tetrapod. This residue lowers the affinity of the human interaction as shown by the Pro_27_ → Ser (Zondlo et al. 2006) or Pro_27_ → Ala mutation (supplementary fig. S7, Supplementary Material online) and contributes to a functional affinity in the range of 100 nM for the human and chicken interactions. Thus, we speculate that the affinity of an ancestral fish/tetrapod complex became too high and was attenuated in the tetrapod lineage by selection of a helix-interfering proline, while in fishes, different changes at position 26 and 27, and also further C-terminally, modulated affinity. Importantly, the Pro_27_ → Ala mutation illustrates how easily the affinity could increase in the human interaction and therefore, by inference, that there is a stabilizing selection for functional affinity rather than for sequence conservation, involving purifying selection against higher affinity. Similar conclusions were reached from experiments in yeast involving poorly conserved disordered regions in MAP kinase signaling (Zarin et al. 2017), for linker regions (González-Foutel et al. 2022) and very recently for coronavirus nucleocapsid protein (Alston et al. 2023).

As a general mechanism, flanking regions can buffer against changes in the binding motif and vice versa, as recently noted for yeast general regulatory factors (Langstein-Skora et al. 2022), and several examples of flanking regions that modulate affinity of motif-mediated interactions have been described (Palopoli et al. 2018; Staby et al. 2021; Theisen et al. 2021; Karlsson et al. 2022; Karlsson et al. 2023). Additionally, it has been observed that the flanking regions of short linear motifs often maintain similar amino acid composition and are more conserved compared to disordered regions without an embedded interaction motif (Chica et al. 2009). Our data suggest that many of the relatively weak interactions observed between binding motifs and interaction domains in general (*K_D_* in the high micromolar–low millimolar range) may be stronger due to nonspecific interactions provided by flanking regions. A picture is emerging where the context in terms of flanking regions and even supertertiary structure (Tompa 2012) proves important for a functional affinity in motif-mediated interactions between short disordered interaction motifs and globular domains (Prestel et al. 2019; Bugge et al. 2020; Laursen et al. 2020; Theisen et al. 2021).

Finally, it is worth emphasizing the dynamic evolution of p53. For example, it is clear that complete gene loss has occurred of either p53_P1_ or p53_P2_ and that several lineages have survived such an event. In this respect, we can say that there is, or at least was, redundance in function between the p53 paralogs. It is also clear from the sequence data that p53TAD is subject to an ongoing evolution and that many types of changes in this disordered region are compatible with function. However, we can only see what works but not what does not function without in vivo or cell-based experiments, which pose an attractive route for future investigation.

## Materials and Methods

### Reconstruction of Ancestral Sequences

We aligned 62 p53TAD sequences, including both paralogs p53TAD_P1_ and p53TAD_P2_, from 51 Actinopterygii species initially using ClustalO (Sievers et al. 2011), followed by manual curation. In uncertain cases, the identity of the respective paralog was confirmed by chromosomal location. This was done by manual inspection of the ENSEMBL (release 104) and NCBI (accessed November 2021) genome browsers. p53TADs from human, *L. chalumnae* and *C. milii* were used as outgroups. In addition to p53TAD, we also aligned 48 MDM2 SWIB domain sequences. Ancestral sequence estimation for both p53TAD and MDM2 SWIB was conducted in MEGAX v10.1.8 (Kumar et al. 2018) using the default JTT + G + I model of sequence evolution (with 5 gamma categories) and the maximum likelihood reconstruction method (see supplementary Spreadsheets S1 and S2, Supplementary Material online and next section for additional details on model selection). The reconstructions used both the sequence alignments and the species phylogeny as input (Fig. 2). We used the time-calibrated phylogenomic tree estimated by Hughes et al. (2018), after pruning out all nontarget species in their tree and manually grafting a few missing species for which we could obtain p53TAD or MDM2 SWIB domain sequences. Missing species grafting followed the phylogenetic placement and ages of these taxa in the fish tree inferred by Rabosky et al. (2018). To accurately reconstruct ancestral sequences for the different p53TAD paralogs examined, the teleost whole-genome duplication event was accounted for by manually duplicating the teleost clade in the phylogenomic tree at 303 Mya, which is within the timeframe estimated for the duplication event by molecular phylogenetic studies (Davesne et al. 2021).

While the species were overall similar in the p53TAD and MDM2 alignments, there were differences in cases where a reliable MDM2 sequence could not be found. For example, we used *Coregonus lavaretus* p53TAD_P2_ and *Coregonus clupeaformis* MDM2 SWIB domain. In other cases, we did not replace the MDM2, thus ending up with fewer species in total. However, extant fish families were well represented in both reconstructions. For MDM2, the N-terminus of reconstructed node 94 was edited due to too few sequences at positions 4 and 5 (gaps were inserted). Due to the uncertainty and for consistency purposes with a previous study (Mihalič et al. 2023), we used expression constructs starting at SQ. In addition, the end of the sequence for reconstructed node 94 is also very uncertain because of a few fish sequences in the alignment. We used low-probability AltAll versions of p53TAD and MDM2_teleost_ to check how robust our experimental results were to errors in the reconstructed sequence, an approach previously described in detail (Eick et al. 2017). For these AltAll variants, we used a cutoff for the posterior probability for the maximum likelihood residue of <0.90 and a probability of the second most likely residue of at least 0.10. All residues that fulfilled these criteria were then included in the AltAll variant (supplementary Spreadsheets S3 and S4 and Text Files S1 and S2, Supplementary Material online). If the affinity of the ML and AltAll variants is similar, it is likely that the true ancestral variant displayed a similar affinity.

### Model Selection for the Reconstruction of Ancestral Sequences

All the analyses on protein sequence reconstruction were performed using the default JTT + G + I model in MEGAX. After all analyses and experiments were conducted, including protein expression and purification and fluorescence polarization, a reviewer raised a concern that we had not provided a rationale for our choice of amino acid substitution model. In response to this feedback, we undertook a substitution model selection procedure in MEGAX for both the MDM2 and p53TAD alignments during the revision process. For MDM2, the JTT + G + I model emerged as the best-fit model, as evidenced by the Akaike information criterion (AIC) scores corrected for a small sample size (AICc: 6783.2; Akaike weights 0.675) (supplementary Spreadsheet S3, Supplementary Material online, second sheet). For the p53TAD alignment, while the default JTT + G + I model ranked 8 according to AIC scores (or third according to BIC scores) among the 56 evaluated models, it received significantly less statistical support compared to the more complex JTT + G + I + F model, which was identified as the best fit (Akaike weights of 9.999 × 10^−01^ vs. 3.879 × 10^−43^ for JTT + G + I) (supplementary Spreadsheet S3, Supplementary Material online, third sheet). Even though it is undeniable that model selection impacts ancestral sequence reconstructions, it is worth noting that the majority of p53TAD reconstructions based on these 2 models exhibit strong similarities, with variations primarily observed in residues where the reconstruction has a high degree of uncertainty and which is covered by AltAll variants for single changes (supplementary Spreadsheet S3, Supplementary Material online, second sheet). This suggests that model selection is unlikely to introduce additional biases into our inferences regarding the evolution of binding affinity for the p53TAD canonical motif in fishes.

### MDM2 SWIB and Full-Length p53TAD Protein Expression and Purification

The cDNA encoding a p53TAD from *D. rerio* with both potential TADs (denoted “longer”; [QNDSQEFAELWEKNLISIQPPGGGSCWDIINDEEYLPGSFDPN] residues 12 to 55 using the numbering for human p53TAD) was ordered in a prSET vector. This resulted in a C-terminally His_6_-Lipo-Thrombin (cleavage site) tagged sequence. The human MDM2 and the *D. rerio* MDM2 were ordered in a pSY10 plasmid that resulted in constructs that were N-terminally NusA-TEV-His_6_-PreScission (cleavage site) tagged. All other MDM2 and full-length p53 proteins used in this study were ordered in pETM33 vector that upon expression produced N-terminally His_6_-GST-PreScission-tagged constructs. The addition of hexa-histidine, Lipo, NusA, or GST tags was used to facilitate higher expression levels and enable simplified protein purification. All sequences were ordered from GenScript (Netherlands) and can be found in supplementary Spreadsheet S5, Supplementary Material online.

All constructs were purified as described previously (Mihalič et al. 2023). In short, *Escherichia coli* BL21 (DE3) cells (Invitrogen) were transformed with plasmid constructs and grown in 2× YT medium at 37 °C until the OD_600_ reached 0.6-0.8. At this point, protein overexpression was induced with the addition of 1 mM isopropyl-β-d-thiogalactopyranoside and protein was expressed overnight at 18 °C. In the case of p53TAD*_D.rerio_*^full-length^, MDM2*_H.sapiens_*, and MDM2*_D.rerio_*, the cells were palleted and resuspended in binding buffer (400 mM sodium chloride, 50 mM sodium phosphate, pH 7.8, and 10% glycerol). Cell lysis was achieved by sonication and the lysate was clarified by centrifugation at 4 °C. The supernatant was filtered and loaded onto a Nickel Sepharose Fast Flow column (GE Healthcare). The column was washed with binding buffer and the proteins of interest were eluted with the binding buffer supplemented with 250 mM imidazole and applied to size exclusion chromatography column Hi load 16/60 Sephacryl S-100 column (GE Healthcare). Fractions of interest were pooled, and the cleavage of tag was induced by the addition of Thrombin or PreScisson proteases overnight at 4 °C. Final run of size exclusion was employed to remove the tag and produce pure protein.

In the case of other constructs cloned into pETM33 vector, protein expression was done in the same manner. Upon sedimentation of bacteria, they were resuspended in binding buffer 2 (50 mM Tris/HCl pH 7.8, 300 mM NaCl, 10-µg/mL DNase I and RNase, 4 mM MgCl_2_, 2 mM CaCl_2_, and cOmplete EDTA-free Protease Inhibitor Cocktail) and sonicated, followed by centrifugation. The supernatant was filtered, applied to Pierce Glutathione Agarose beads (Thermo Scientific), washed with wash buffer (50 mM Tris, 300 mM NaCl, and pH 7.8), and eluted with wash buffer supplemented with 10 mM reduced glutathione. Eluted fractions were cleaved overnight with PreScission protease to remove the GST tag and the tag was removed by applying of the cleavage reaction onto the Nickel Sepharose Fast Flow column. Flow through was collected and the final polishing step was performed using a Hi load 16/60 Sephacryl S-100 column. Sample purity after the final step of purification was assessed by SDS–PAGE and matrix-assisted laser desorption/ionization time-of-flight mass spectrometry and samples were dialyzed against experimental buffer (20 mM sodium phosphate, pH 7.4, 150 mM NaCl, and 1 mM TCEP). Protein concentration was determined by absorbance at 280 nm and samples were frozen at −80 °C until further use. Peptides corresponding to p53TAD^15–27^ were purchased from GeneCust (France) in N-terminal acetylated form and dissolved in an experimental buffer. The concentration of the peptides was determined by measuring A_280_.

### Fluorescence Polarization Experiments

The affinity between p53TAD and its corresponding MDM2 was determined by a fluorescence polarization assay. First, the affinity between FITC-labeled p53TAD_human_^15–26^ and all MDM2s was determined by keeping FITC-p53TAD_human_^15–26^ at low concentration (15 nM) and measuring FP at increasing concentrations of MDM2. A 1-site binding model was fitted to these data to obtain *K*_D_ for FITC-p53TAD_human_^15–26^ for each MDM2 variant. Next, to determine the affinity for native and nonnative interactions, a complex between MDM2 (0.2 μM for *H. sapiens*; 0.5 μM for Actinopterygii AltAll; 1 μM for *Lepisosteus oculatus*, *A. anguilla*, *C. harengus*, *D. rerio*, teleost ML, and teleost AltAll; 1.5 μM for *C. Argus*, *D. rerio*, and Actinopterygii ML; and 2 μM for *Maylandia zebra*, respectively) and FITC-p53TAD_human_^15–26^ (15 nM) was displaced by increasing concentrations of various short p53TAD^15–27^ peptides or p53TAD^full-length^. From the displacement curve and *K_D_* for FITC-p53TAD_human_^15–26^, we calculated *K_D_* values for the native and nonnative interactions for “displacer” peptides as described by Nikolovska-Coleska et al. (2004) (supplementary Spreadsheet S6, Supplementary Material online). Parameters from binding experiments were obtained by curve fitting in GraphPad Prism 9.

### Isothermal Titration Calorimetry

For ITC experiments, a MicroCal iTC200 instrument (GE Healthcare) was used, and the experiments were performed at 25 °C. The MDM2 protein and p53TAD peptides were dialyzed against the experimental buffer prior to experiments to avoid buffer mismatch. The concentration of *D. rerio* MDM2 in the cell was 11 μM and the concentration of p53TAD_*D.rerio*_^17–32^ and p53TAD_*D.rerio*_^10–75^ in the syringe was 118 and 116 μM, respectively. Results were analyzed using the built-in software and a 2-state binding model was assumed.

## ColabFold Predictions

We used ColabFold (Mirdita et al. 2022) to predict the interactions between p53TAD^15–27^ peptides or p53TAD^full-length^ and the respective native MDM2. Only the highest confidence model was analyzed (supplementary fig. S9, Supplementary Material online).

## Supplementary Material

msae018_Supplementary_Data

## Data Availability

The data underlying this article are available in the article and in its online Supplementary material. A Prism file with *K_D_* calculations is available upon request (it is not accepted as Supplementary material).
