# Supplementary material for: Conservation of Affinity Rather Than Sequence Underlies a Dynamic Evolution of the Motif-Mediated p53/MDM2 Interaction in Ray-Finned Fishes
Source: Mol Biol Evol. 2024 Feb 1;41(2):msae018. doi: 10.1093/molbev/msae018 (PMC10901556; doi:10.1093/molbev/msae018)
Supplement: msae018_Supplementary_Data [file msae018_supplementary_data.zip › Mihalic_et_al_Supplementary_Information_v6.pdf]

Supplementary information

Conservation of affinity rather than sequence underlies a dynamic evolution of the motif-mediated p53/MDM2 interaction in ray-finned fishes

Filip Mihalič[1]*, Dahiana Arcila[2], Mats E. Pettersson[1], Pouria Farkhondehkish[1], Eva Andersson[1], Leif Andersson[1, 3], Ricardo Betancur-R.[2] and Per Jemth[1]*

[1]Department of Medical Biochemistry and Microbiology, Uppsala University, BMC Box 582, SE-75123 Uppsala, Sweden.

[2] Scripps Institution of Oceanography, University of California San Diego, La Jolla, CA 92093, U.S.A.

[3]Department of Veterinary Integrative Biosciences, Texas A&M University, College Station, USA

*Corresponding author: Filip Mihalič, e-mail: filip.mihalic24@gmail.com

*Corresponding author: Per Jemth, e-mail: per.jemth@imbim.uu.se

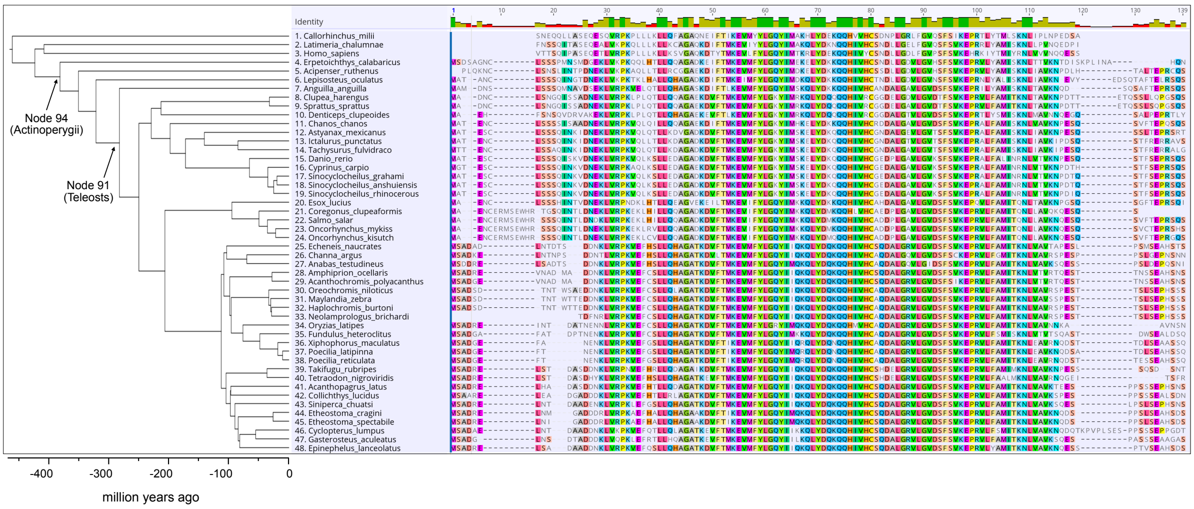

Figure S1. Sequence alignment of the SWIB domain from MDM2. The sequence alignment was used for reconstruction of ancestral fish MDM2 SWIB domains.

MEGA X maximum likelihood reconstruction of residues 24-27 in $p53TAD_{P1}$

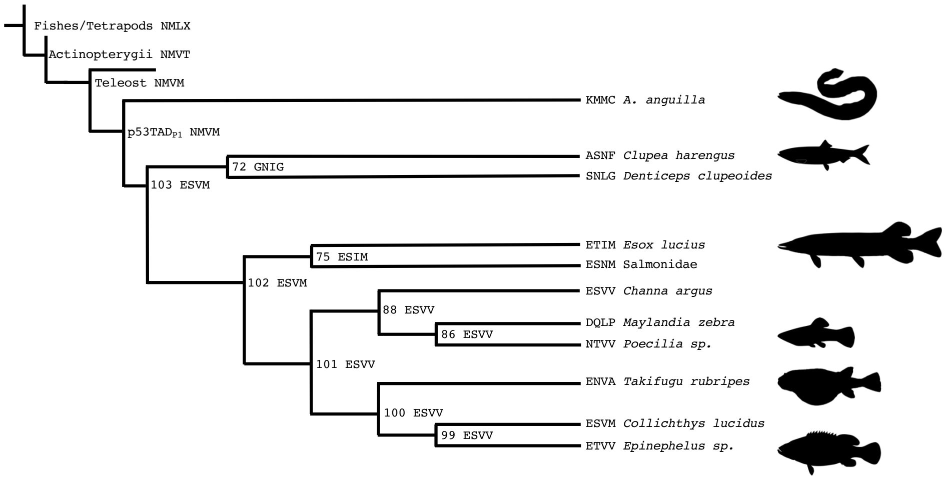

Figure S2. Evolution of $p53TAD_{P1}^{24\text{-}27}$. The maximum likelihood residues for residues 24-27 for nodes in the phylogenetic tree in Fig. 2 together with selected extant sequences. See Supplementary Spreadsheet 2 and 3 and Supplementary text file 1 for posterior probabilities and AltAll residues. Pictures are from Phylopic.org.

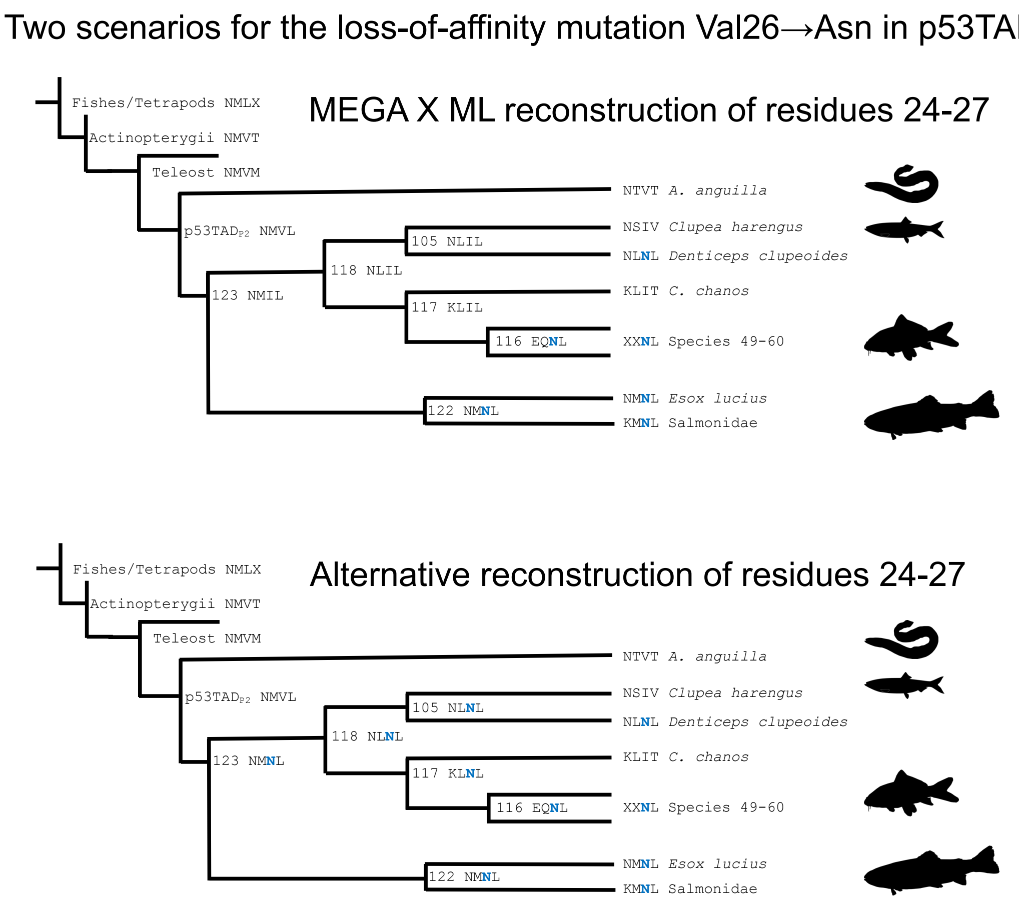

Figure S3. Evolution of p53TAD$_{P2}$$^{24\text{-}27}$. The maximum likelihood residues for residues 24-27 for nodes in the phylogenetic tree in Fig. 2 together with selected extant sequences. See Supplementary Spreadsheet 2 and 3 and Supplementary text file 1 for posterior probabilities and AltAll residues. Two different scenarios are shown, (*i*) the one reconstructed by MEGAX where the first mutation is Val$_{26}$→Ile, and where Asn (blue N) is introduced later at three different occasions, and (*ii*) an alternative scenario where Val$_{26}$→Asn occurs already at node 103 and Ile$_{26}$ is introduced later in separate lineages. In both cases convergence is observed, suggesting that certain (combinations of) residues are preferred for optimal fitness. Pictures are from Phylopic.org.

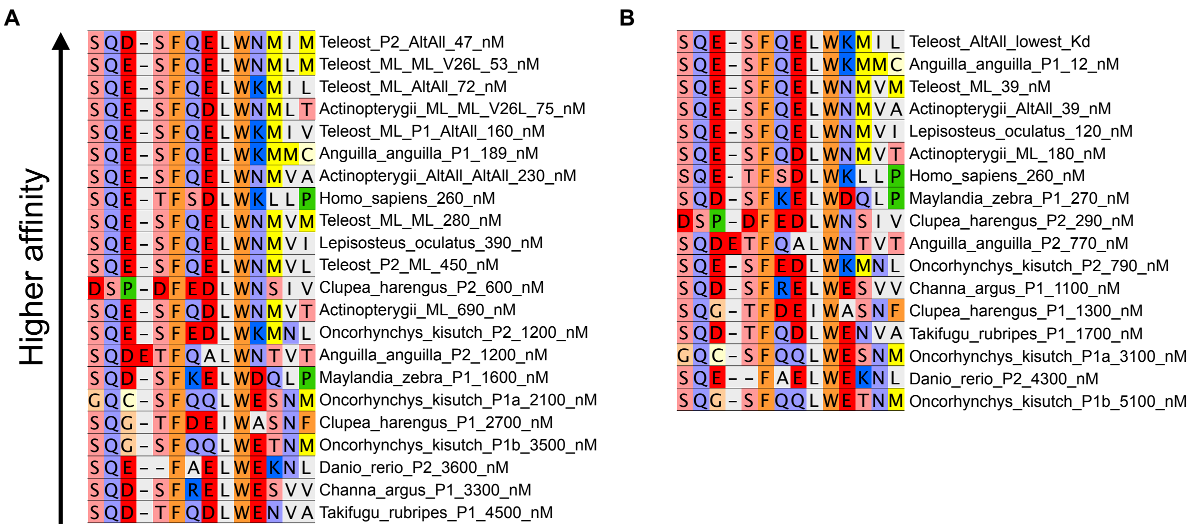

Figure S4. Sequence alignment by affinity. The p53TAD peptides with highest affinity are at the top and those with lowest at the bottom. The order is based on (A) affinity for the native MDM2 and (B) affinity for human MDM2.

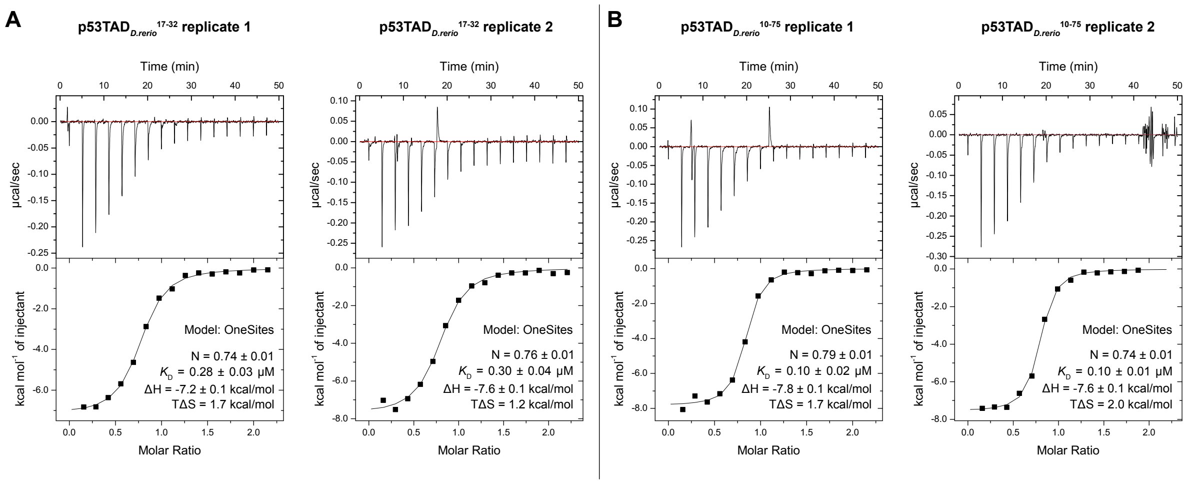

Figure S5. ITC measurement of the interaction between *D. rerio* MDM2 and p53TAD. (A) Interaction between MDM2 and short peptide p53TAD$_{D.rerio}^{17\text{-}32}$. (B) Interaction between MDM2 and full length p53TAD$_{D.rerio}^{10\text{-}75}$. Two replicates of each experiment were performed.

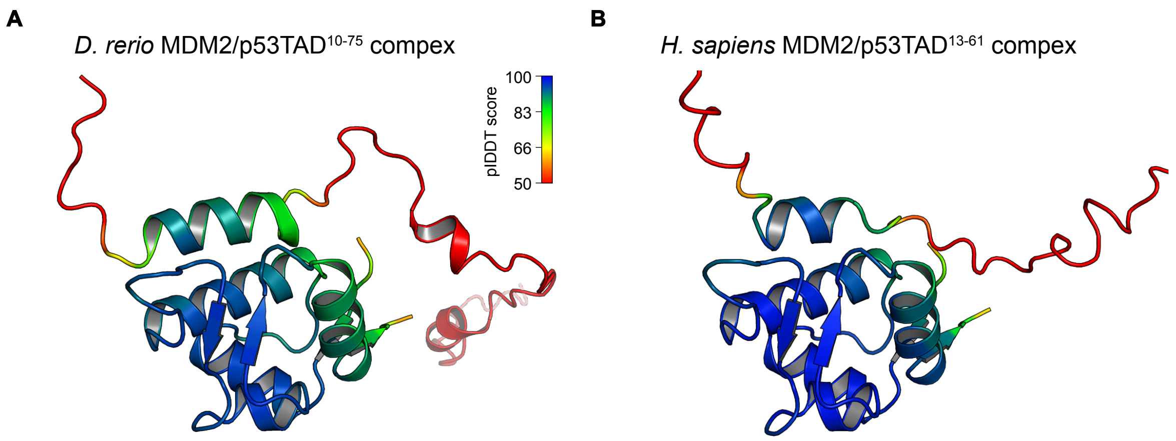

Figure S6. Colabfold prediction of the MDM2/p53TAD$^{Full\ length}$ complexes. A) Prediction for *D. rerio* complex. Models are colored in spectra in accordance with the prediction confidence score (pIDDT) with blue signifying high prediction confidence and red low confidence (pIDDT<50). **B)** prediction for *H. sapiens* complex. Coloring as in B). Note a significantly shorter helix of the bound p53 binding motif for the human complex in comparison to *D. rerio* complex.

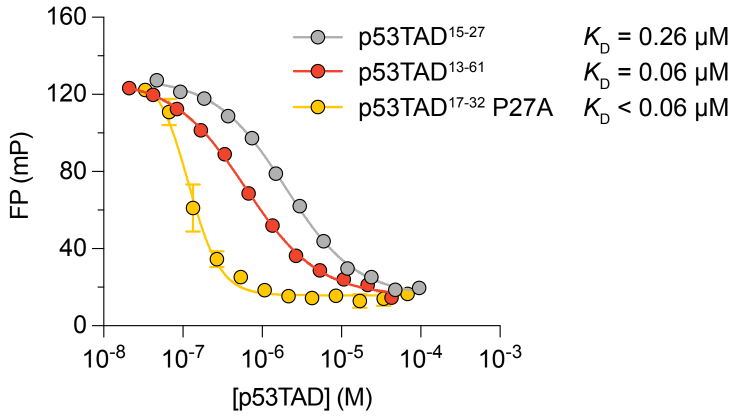

Figure S7. High affinity of human p53TAD$^{17-32}$ P27A mutant. Fluorescence polarization experiment showing displacement of the labeled probe by human p53TAD$^{15-27}$ (short), p53TAD$^{13-61}$ (full length) and p53TAD$^{17-32}$ P27A mutant. Note that the K_D of the p53TAD$^{17-32}$ P27A mutant is too low to be determined in our fluorescence polarization assay, likely in the low to sub nM range.

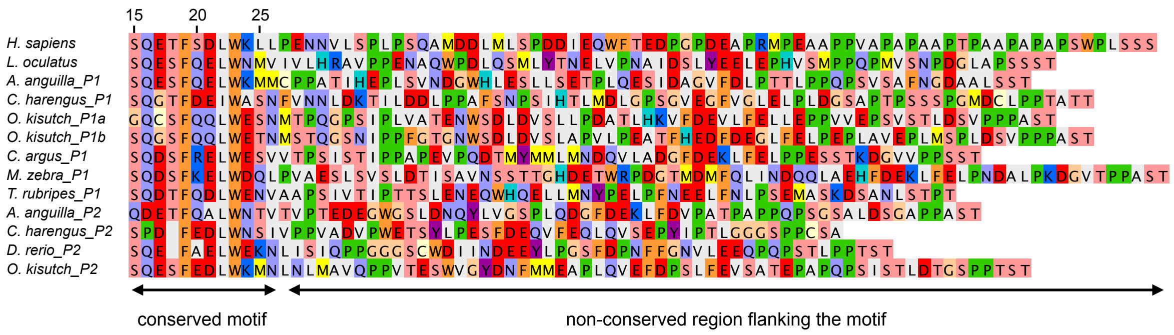

Figure S8. Non-conserved nature of the C-terminal flanking region. The transactivation domains of extant fish p53TAD constructs used in this study were aligned at the conserved binding motif (without the N-terminal part). The C-terminal flanking regions are "aligned" without gaps to showcase the variability and lack of clear patterns in the sequence following the conserved Trp_{23}. Note that the motif with the highest affinity (*A. anguilla* P1) has methionine both at position 25 and 26.

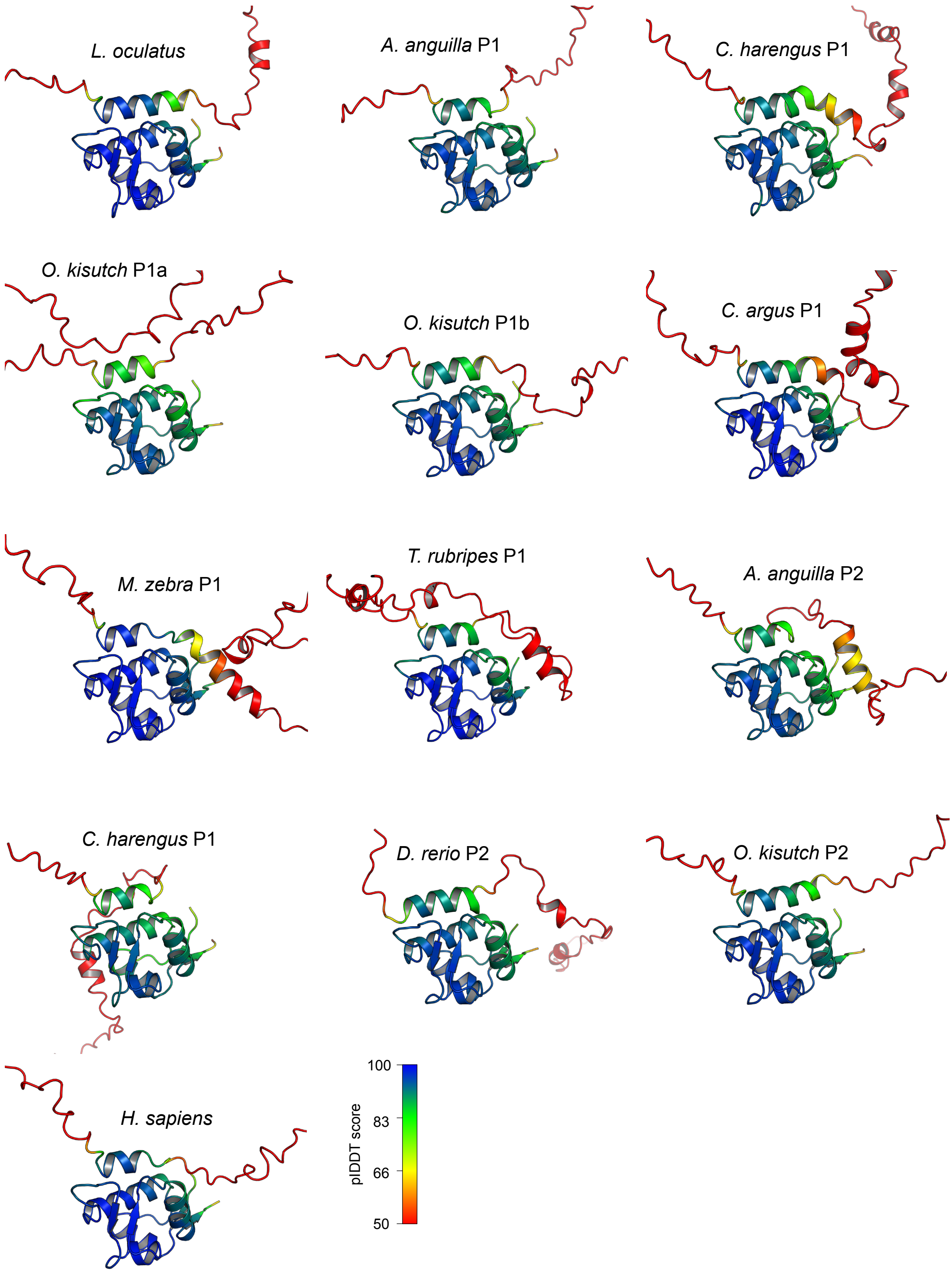

Figure S9. Colabfold prediction for all present day MDM2/p53TAD$^{\text{full length}}$ interactions investigated in this study. The structures are colored according to the confidence score of the prediction, blue is highest confidence and red lowest.

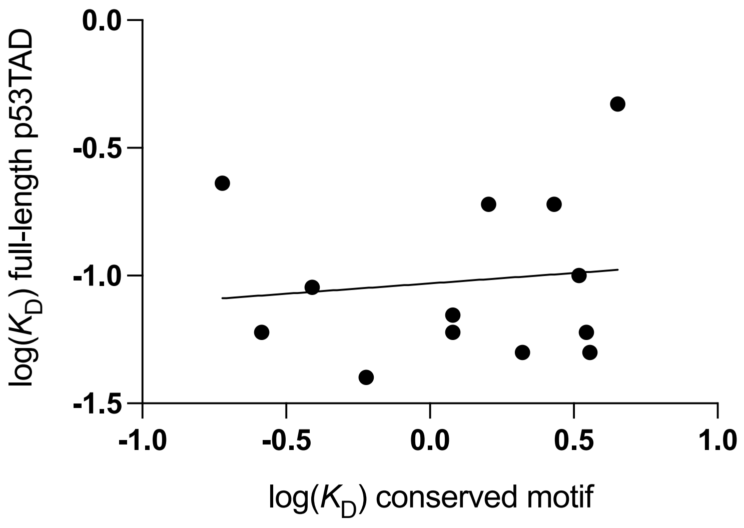

Figure S10. Correlation of K_D values for the short, conserved interaction motif and full-length p53TAD. Data for extant fish and human p53TAD/MDM2 interactions are plotted on a log scale to reflect the correlation of free energy of binding, ΔG (R^2=0.014). On a linear scale, R^2 is slightly higher (0.15), but still very low. The poor correlation is consistent with a role of the flanking region in mitigating the affinity between the proteins.

Supplementary Spreadsheet 1. Ancestral sequence reconstruction of nodes in the p53TAD tree (**Fig. 2**).

Supplementary Spreadsheet 2. Ancestral sequence reconstruction of nodes in the MDM2 SWIB tree (**Fig. S1**).

Supplementary Spreadsheet 3. Reconstructed maximum likelihood (ML) and AltAll sequences for p53TAD used in the study. The number in bold refers to the node in the tree and other numbers are posterior probabilities from the reconstruction (**Supplementary Spreadsheet 1**). The second and third sheets provide data on evolutionary model comparison.

Supplementary Spreadsheet 4. Reconstructed maximum likelihood (ML) and AltAll sequences for MDM2 SWIB domain used in the study. Residue numbers, node numbers and posterior probabilities from the reconstruction (**Supplementary Spreadsheet 2**) are shown. The second sheet provides data on evolutionary model comparison.

Supplementary Spreadsheet 5. Sequences of p53TAD peptides and MDM2 SWIB domains used in the study.

Supplementary Spreadsheet 6. Calculated K_D from fluorescence polarization displacement experiments.

Supplementary Table 1. Affinities between ancestral p53TAD$^{15-27}$ and MDM2. The complex between labeled $p53TAD_{Human}^{15-27}$ and reconstructed $MDM2_{Actinopterygii}$ or $MDM2_{Teleost}$ was displaced by unlabeled reconstructed p53TAD$^{15-27}$ from the different evolutionary nodes. Native maximum likelihood K_D values are in bold.

**Ancestral species (node)**[a]	**p53TAD$^{15-27}$** Reconstructed	**K_D (μM)**[b] Native	**K_D (μM)**[c] MDM2 *H.sapiens*	**K_D (μM)**[d] p53TAD *H.sapiens*
Actinopterygii (Ray-finned fishes) (128) p53TAD^{ML}/MDM2^{ML}	SQESFQDLWNMVT	**0.69±0.05**	0.18±0.007	0.40±0.03
Actinopterygii p53TAD^{ML}/MDM2^{ML} Val_{26}→Leu	SQESFQDLWNMLT	0.075±0.012		
Actinopterygii p53TAD^{AltAll}/MDM2^{ML}	SQESFQELWNMVA	0.46±0.02	0.039±0.004	
Actinopterygii p53TAD^{ML}/MDM2^{AltAll}	SQESFQDLWNMVT	0.35±0.05		0.27±0.01
Actinopterygii p53TAD^{AltAll}/MDM2^{AltAll}	SQESFQELWNMVA	0.23±0.01		
Teleost (125) p53TAD^{ML}/MDM2^{ML}	SQESFQELWNMVM	**0.28±0.02**	0.039±0.01	0.72±0.08
Teleost p53TAD^{ML}/MDM2^{ML} Val_{26}→Leu	SQESFQELWNMLM	0.053±0.01		
Teleost p53TAD^{AltAll}/MDM2^{ML}	SQESFQELWKMIL	0.072±0.008	Beyond detection limit, low to sub nM)	
Teleost p53TAD^{AltAll2}/MDM2^{ML}	SQESFQELWKMIV	0.16±0.02		
Teleost p53TAD^{ML}/MDM2^{AltAll}	SQESFQELWNMVM	0.21±0.01		0.64±0.1

Teleost[e] p53TAD^{AltAll1}/MDM2^{AltAll}	SQESFQELWKMIL	0.10±0.02		
Teleost[e] p53TAD^{AltAll2}/MDM2^{AltAll}	SQESFQELWKMIV	-		
Teleost (104/125) p53TAD$_{P1}^{ML}$/MDM2^{ML}	SQESFQELWNMVM	**0.28±0.02**		
Teleost[e] p53TAD$_{P1}^{AltAll1}$/MDM2^{ML}	SQESFQELWKMIL	0.072±0.008		
Teleost[e] p53TAD$_{P1}^{AltAll2}$/MDM2^{ML}	SQESFQELWKMIV	0.16±0.02		
Teleost p53TAD$_{P1}^{ML}$/MDM2^{AltAll}	SQESFQELWNMVM	0.21±0.01		
Teleost[e] p53TAD$_{P1}^{AltAll1}$/MDM2^{AltAll}	SQESFQELWKMIL	0.10±0.02		
Teleost[e] p53TAD$_{P1}^{AltAll2}$/MDM2^{AltAll}	SQESFQELWKMIV	-		
Teleost (124/125) p53TAD$_{P2}^{ML}$/MDM2^{ML}	SQESFQELWNMVL	**0.45±0.01**		
Teleost p53TAD$_{P2}^{AltAll}$/MDM2^{ML}	SQDSFQELWNMIM	0.05±0.03		

[a]Node refers to the node number in the reconstruction in **Figure 2** and **Supplementary Spreadsheet 1**.
[b]Native K_D refers to the interaction between reconstructed p53TAD and MDM2 variants from the corresponding nodes in the phylogenetic trees (**Fig. 2** and **Supplementary Fig. S1**).
[c]K_D for the interaction between MDM2$_{H.sapiens}$ and the respective reconstructed p53TAD peptide.
[d]K_D for the interaction between p53TAD$_{H.sapiens}$ and the respective reconstructed MDM2 variant.
[e]MDM2$_{Teleost}$ was used in experiments with all three reconstructed p53TAD variants: p53TAD$_{Teleost}$, p53TAD$_{P1}$ and p53TAD$_{P2}$ since only one copy of MDM2 was found in modern teleost fishes. Thus, the reconstructed MDM2 is the same for nodes 104, 124 and 125 in the p53TAD tree. Furthermore, the two ML and AltAll variants for p53TAD$_{Teleostei}$ and p53TAD$_{P1}$ are identical (**Supplementary Spreadsheet 3)**.
